# Thoracoscopic resection for esophageal cancer: A review of literature

**DOI:** 10.4103/0972-9941.38909

**Published:** 2007

**Authors:** Joris J G Scheepers, Donald L van der Peet, Alexander A F A Veenhof, Miguel A Cuesta

**Affiliations:** Department of Surgery, Vrije Universiteit Medical Centre (VUMC), Amsterdam, Netherlands

**Keywords:** Cancer, esophagus, laparoscopy, thoracoscopy

## Abstract

Esophageal resection remains the only curative option in high grade dysplasia of the Barrett esophagus and non metastasized esophageal cancer. In addition, it may also be an adequate treatment in selected cases of benign disease. A wide variety of minimally invasive procedures have become available in esophageal surgery. Aim of the present review article is to evaluate minimally invasive procedures for esophageal resection, especially the approach performed through right thoracoscopy.

## INTRODUCTION

Esophagus resection may be the adequate treatment in selected cases of benign esophageal diseases, but the most frequent indications for esophageal resection are the high grade dysplasia of the Barrett esophagus and the non metastasized esophageal cancer.

Prognosis of patients with esophageal cancer remains poor: only 56% of the patients presented with esophageal cancer have resectable disease with an overall five-years survival rate of 10% while the five-years survival rate among operated patients is still only 18%.[1] The only curative therapy remains surgery. For years the procedure of choice was the Ivor-Lewis operation, later modified by McKeown, in which the tumor is resected through a right-sided thoracotomy in combination with a laparotomy with cervical esophagogastric anastomosis.[2,3] The advantage of this operation is the perfect exposure allowing complete esophageal dissection and possible en bloc resection.[4,5] Disadvantages may be the pulmonary complications related to the necessary thoracotomy and the collapse of the right lung. Pulmonary complications can be overcome by the transhiatal approach as described by Orringer in which the esophagus is dissected free through the enlarged hiatus and after esophageal-proximal gastric resection the created gastric tube is anastomosed with the cervical esophagus through a combined cervico-abdominal approach, avoiding a thoracotomy.[6] Disadvantage of this approach is the partly blind dissection of the esophagus and the tumor and its limitation to tumors of the distal esophagus. Both procedures have high complication rates, varying from 40 to 80% and in-hospital mortality varying from an average of 7.5 % to below 5% in experienced centers.[5]

There is still controversy about the approach and extent of necessary resection. A recent prospective randomized study, in which en bloc esophageal resections with systematic abdominal and mediastinal lymph node dissection (two fields lymphadenectomy) has been compared with the classical transhiatal approach, showed that the transhiatal approach carries lower morbidity than through right thoracotomy performed extended en-bloc lymphadenectomy. Moreover, a trend was observed with advantage for the transthoracic approach in tumors located in the mid-esophagus, for the most frequent lower esophageal cancer the median survival, disease free and quality-adjusted survival were not statistically different.[7]

In an attempt to lower the mortality and morbidity rates of the conventional esophagectomy, advantages in minimally invasive instrumentation and gained experience in minimally invasive surgery make a minimal invasive approach possible. Several minimal invasive approaches mimic the conventional procedures for esophagectomy: 1) transhiatal esophageal resection by laparoscopy; 2) esophagectomy by right thoracoscopy, the so called three-stage operation and 3) esophageal resection by means of endoscopic microsurgical mediastinal dissection.[8–11] Moreover, transhiatal and thoracoscopic esophageal resection have also been performed endoscopically with assistance of the robot.[12–13]

The aim of the present review article is to evaluate this minimal invasive approach for esophagus resection, especially the approach performed through right thoracoscopy.

## INDICATIONS FOR MINIMAL INVASIVE ESOPHAGEAL RESECTION

Benign esophageal diseases are an infrequent indication for esophageal resection. Nevertheless, important caustic and peptic stenosis not suitable for treatment by balloon dilatation may finally be considered indications for resection. End stage motility diseases of the esophagus like achalasia and Chagas's disease with important mega-esophagus and the presence of multiple esophageal epiphrenic diverticula may also be an indication for resection. Also, borderline high grade dysplasia of the Barrett esophagus may be a good indication for resection by minimal invasive techniques.

Cancer of the esophagus is the most frequent indication for resection. Once diagnosed, it is important to establish a good preoperative assessment of the resectability. Enhanced CT scan of thorax and abdomen and an endoscopic ultrasonography can determine the lymph node involvement, local growth in other organs and existence of metastases. Positron emission tomography (PET's scan) is currently under evaluation in order to assess the capacity to properly exclude distant metastases. Moreover, treatment of the esophageal cancer needs a multidisciplinary approach and usually patients with a locally advanced cancer will be treated by neo-adjuvant chemo-radiation, making surgery perhaps more difficult.

All operable cancers in any location can be approached by minimal invasive surgery. For distal tumors the transhiatal or the right thoracoscopic approaches seem both a good option whereas for higher tumors, in the proximal esophagus and around the carina the right thoracoscopic approach is the only possibility.

### Different minimally invasive procedures

1. Resection of the esophagus (and lymphadenectomy) through a right thoracoscopy. Mobilization of the stomach and creation of gastric conduit can be performed by laparotomy or by laparoscopy followed by a cervical anastomosis (three stage technique).
2. Ivor Lewis operation: right thoracoscopy (esophagectomy and lymphadenectomy) followed by laparoscopic gastric mobilization and intrathoracic anastomosis between mediastinal esophagus and gastric tube.
3. Transhiatal approach, total laparoscopic or laparoscopic assisted dissection of the esophagus up to the carina followed by mobilization of the stomach, creation of the gastric conduit and subsequent cervical anastomosis.
4. Esophageal resection through mediastinoscopy: endoscopic microsurgical dissection.
5. Robot assisted procedures

## RIGHT THORACOSCOPIC AND THREE STAGE PROCEDURE

From a historical point of view, Dallemagne *et al.*, described in 1992 the first combined thoracoscopic and laparoscopic approach for esophageal resection for cancer with reconstruction by means of a gastric conduit anastomosed to the neck[14] and in the book ‘Minimally Invasive Surgery in Gastrointestinal Cancer’ (Cuesta and Nagy, editors. 1993) described the outcome of the first twelve patients operated on in this way during the period between June 1991 to July 1992.[15] Six tumors were located in the midesophagus and another six in the lower esophagus; seven were squamous cell cancers and five were adenocarcinomas. In 2 of the 12 patients, the abdominal stage was laparoscopically performed. The operative times for these procedures were nine and seven hours respectively and the mean operative time for the thoracoscopic stage was 2.3 hours (range 2 to 3.15 hours). One postoperative death occurred because myocardial infarction and another three patients died within 30 postoperative days because of complications related to partial gastric tube necrosis. The author could not explain this problem because it had not been encountered with the traditional technique.

In the same year, Azagra *et al.* and Cuschieri *et al.*, described their initial experience with a small series of patients with esophageal cancer in whom the esophagus resection and mediastinal lymphadenectomy was performed through a right thoracoscopic approach.[16–17] The rest of operation was completed through a laparotomy and consequent cervical approach. Mean times for the thoracoscopic approach were between three and fours hours. Gossot *et al.*, performed the same thoracoscopic esophageal resection in 15 patients in 1993, had 20% conversion rate (large tumor in one patient and incomplete lung collapse in another two) and significant postoperative pulmonary complications in another two patients.[18]

The philosophy for the development of this minimally invasive approach was to obtain the same oncological outcome as the conventional procedure but with all postoperative advantages of the minimally invasive approach. However, the reports of these initial pioneers were followed by others who were critical about the procedure and others who were especially disappointed because of the high rate of postoperative respiratory complications.

The attempt of Cuschieri *et al.*, to change the thoracoscopic approach from a lateral to a prone position, without total collapse of the lung, was designed to diminish the postoperative respiratory complications.[19] The high rate of postoperative pulmonary complications were also found by McAnena *et al.*, (9 patients), Gossot *et al.*, (29 patients) and Dexter *et al.*, (24 patients). They showed comparable results: conversions were between 10-17%, morbidity, especially respiratory, between 17-42% and mortality between 3 and 12%. Leakages and laryngeal nerve palsy were comparable with the conventional approach, 17 and 10%, respectively. They concluded that the technique is feasible, but these initial results do not show a real benefit of the thoracoscopic approach, potential complications remains high and requires further study.[20–22] The same conclusions were drawn by Robertson *et al.*, Peracchia *et al.* and Law *et al.*[23–25]

In spite of these “no advantages” reports, a new impulse for the endoscopic approach of the esophageal resection came from Japanese centres and from Luketich and coworkers from Pittsburgh.

Akaishi *et al.*, performed en bloc total esophagectomy with radical lymphadenectomy in 39 patients with esophageal cancer by right thoracoscopy, hereafter the rest of the operation was performed by conventional means. There were no conversions, operating time was 200 min (± 41 min), blood loss 270 cc (± 157 cc), lymph nodes harvested 19.7 (±11), no mortality and more important the fact that 22 patients of 39 did not require postoperative respiratory support showing only a slight decrease of vital capacity from 100 to 85%. That the decline in pulmonary function was significantly less than in the open technique was an important result of this study.[26] The same conclusions were obtained by Kawahara *et al.*, (23 patients).[27] Osugi *et al.*, described their experience with the three field lymphadenectomy. They compared 77 patients with squamous cell cancer approached by a minithoracotomy and four ports with a control group of 72 patients approached conventionally in a three stage procedure. Retrieved lymph nodes (33 vs. 32 nodes), longer thoracic operation time (227 versus 186 min), less vital capacity reduction (less in the thoracoscopy group 15% vs. 22%, *P*=0, 01) and a similar three and five years survival: 70 and 55% compared with 60 and 57% for the open procedure were remarkable. Their conclusion was that thoracoscopic resection provided comparable results to open radical esophagectomy with less surgical trauma.[28]

The same Japanese group explained the importance of the learning curve in reducing operating time and obtaining better outcomes with this approach. They compared the outcomes of the first 34 operated patients with the last 46 patients. Concerning blood loss, duration of thoracoscopic time, incidence of postoperative respiratory complications and number of retrieved lymph nodes were all significant better in the last group.[29] These results were confirmed by Teguchi *et al.* and Smithers *et al.*, in Australia with a consecutive series of 162 patients during a six-year period.[30,31]

Interesting are the publications of different Japanese groups with operative technical contributions used to reduce the operating time and the surgical stress of the operating surgical team while obtaining the same oncological results. Contributions such as the combination of thoracoscopy and mediastinoscopy by Mafune *et al.*, Ikeda *et al.* and recently described by Okushiba *et al.*, in order to preserve the recurrent nerve function and perform an adequate lymphadenectomy, are remarkable. Hand-assisted thoracoscopic procedures have also been reported in which the surgeon or the assistant introduced one hand in the upper abdominal quadrant through the anterior phrenico-mediastinal route to the right thoracic cavity.[32–34] The hand can be used to separate the lung, visualize the anatomy and help to discriminate the structures. But the most important contribution has been made by Luketich *et al.* Their first publication in 1998 reported the first eight patients[35] and in 2003 their group reported 222 patients operated between 1996 and 2002.[10] Indications included high grade dysplasia (n=47) and esophageal cancer (n=175). Initial experience included 8 transhiatal procedures followed by 214 three-stage approach in which right thoracoscopy and laparoscopy were performed. Non emergent conversion to open procedure was required in 16 patients (7.2%), being the minimal invasive esophagectomy (MIE) successfully completed in 206 patients (92.8%). Pyloromyotomy was performed in 28 patients and pyloroplasty in 136 patients. The use of a narrow gastric tube of 3-4 cm size without pyloroplasty in order to prevent biliary reflux resulted in more leakages and was soon abandoned. Median intensive care stay was one day (range 1-30); hospital stay seven days (Range 3-75). They reported major and minor complication rates of 32% and 23.9%, respectively. Major complications included an anastomotic leak in 26 patients (11.7%), chylothorax in 7 (3.2%), pneumonia in 17 (7.7%) and vocal cord palsy in eight patients (3.6%). Moreover, operative mortality was 1.4% (n=3). At a mean follow-up of 19 months (range 1-68 days) quality of life scores were similar to preoperative values and population norms. Stage specific survival was similar to open series, stage I of 70%, stage II of 20% and III of 25% after 40 months. The results of Nguyen *et al.*, with 46 consecutive patients (cancer in 38 patients, high dysplasia in three and esophageal stricture in five) were concordant with the results obtained by Luketich (mortality 4.3%; hospital stay, eight days; and three-year survival of 57%).[36]

## OPERATIVE TECHNIQUE OF THE THREE STAGE OPERATION

**Figure 1:**
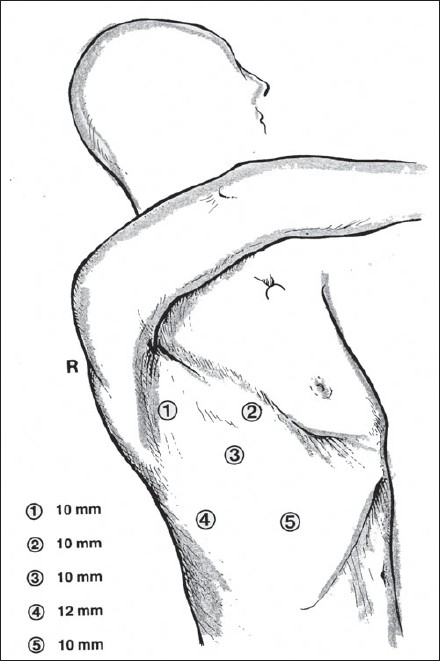
Placement of ports for right thoracoscopic stage of the procedure

**Figure 2:**
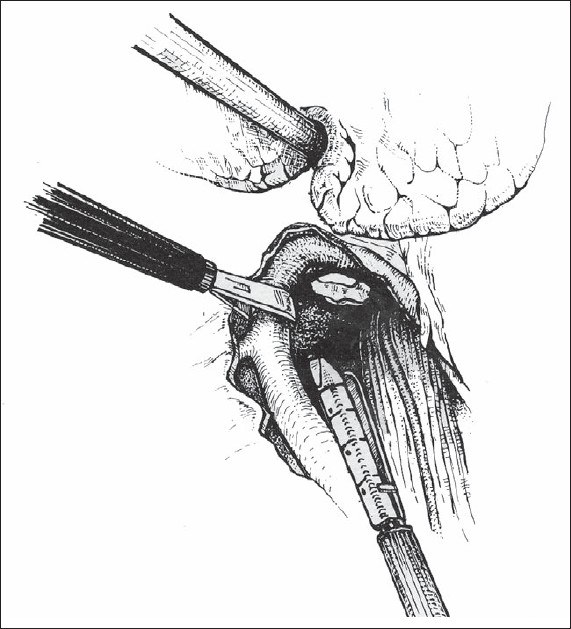
After division of the pleura, the azygos vein is dissected free and divided

**Figure 3:**
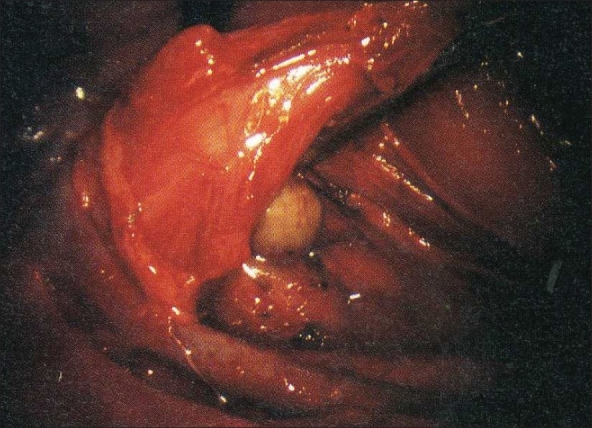
The esophagus, along with lymph nodes and peri-esophageal tissues, is dissected free

The patient is intubated with a double-lumen tube to block the right lung and is positioned in the left lateral decubitus position. The surgeon stands on the right side and assistant on the left. Four or five thoracoscopic trocars are introduced between the anterior and posterior axillary lines [Figure 1]. Luketich *et al.*, placed the 10 mm camera at the 7^th^ to 8^th^ intercostal space, just anterior to the midaxillary line. A 5 mm port is placed at the 8^th^ or 9^th^ intercostal space, posterior to the posterior axillary line for the Ultracision device (Johnson and Johnson). Another 10 mm port is placed in the anterior axillary line at the 4^th^ intercostal space (for a fan to retract the lung) and the last 5 mm port is placed just posterior to the scapula tip (for traction and countertraction instruments)[10]. After division of the inferior pulmonary ligament and after incision of the pleura, a normal segment of the esophagus is mobilized and a Penrose drain is placed around it. The pleura are then divided up to the level of the azygos vein and the vein is divided by endovascular stapler [Figure 2]. The pleura opening continues up to 2-4 cm above the carina. The esophagus is mobilized with the fat tissue and lymph nodes around it up to the planes of aorta, pericardial sac and contralateral pleura [Figure 3]. Because it is not an en bloc resection, the azygos vein and the thoracic duct are not resected. During the division of the tissue around the esophagus, especially the vessels coming from the aorta have to be clipped in order to avoid lymph leakage. The distal part of the esophagus around the hiatus is not dissected during the thoracoscopic stage in order to avoid leakage of CO2 in the chest during the laparoscopic part of the operation.

After drainage of the thoracic cavity patient is turned to the supine position.

**Figure 4:**
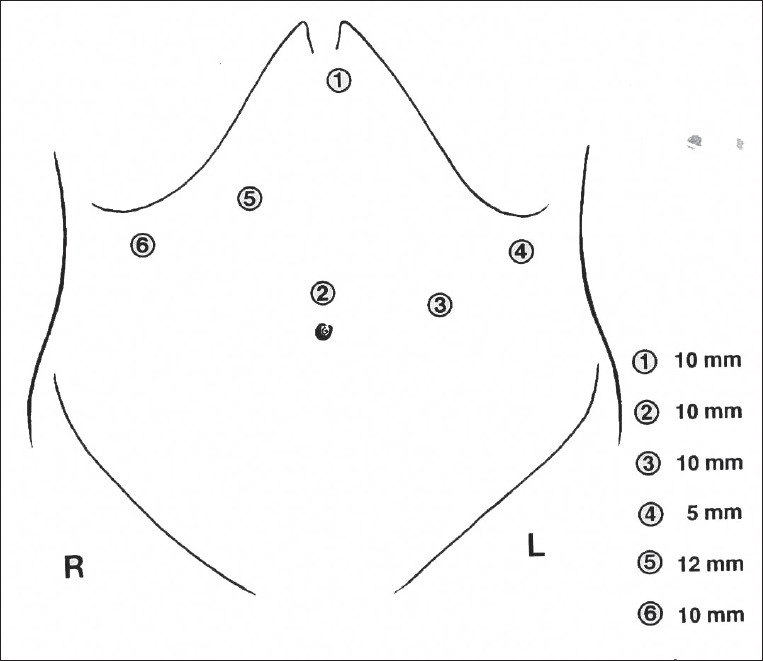
Placement of ports for the laparoscopic stage of the procedure

Left side of the neck is also prepared for the dissection of the esophagus and posterior anastomosis. Surgeon stands between the legs of the patient looking at two monitors placed on the shoulder level of the patient. Five (or six) trocars are introduced in the upper abdomen [Figure 4].

The stomach is dissected free with preservation of the gastroepiploic vessels by means of the Ligasure^®^ or the Ultracision devices.

Once the lesser and greater curvature is dissected up to the hiatus, the stomach is tilted and the left gastric vessels are dissected free and divided by means of Ligasure^®^ device or endostaplers after a lymphadenectomy of the celiac trunk is performed. From there, the rest of the stomach is dissected free up to the hiatus. Controversy exists regarding the pyloroplasty or pyloromyotomy for drainage of the pylorus after creation of a gastric conduit. Our group does not consider the pyloroplasty in any case whereas Luketich *et al.*, does perform it always. A gastric tube of 4 cm is created by means of endostapler 4.8 mm device from the lesser curvature to the fundus of the stomach. Both the proximal part (esophagus and fundus) are fixed by a couple of stitches to the tip of the gastric tube and carefully mobilized with the specimen from the cervical incision (that had been dissected already by a second team [Figure 5, A and B]. After exteriorization of the gastric tube at the cervical wound, it is anastomosed with the proximal esophagus. A jejunostomy feeding catheter is placed laparoscopically and operation finished after closure of the cervical wound, which is drained.

**Figure 5A:**
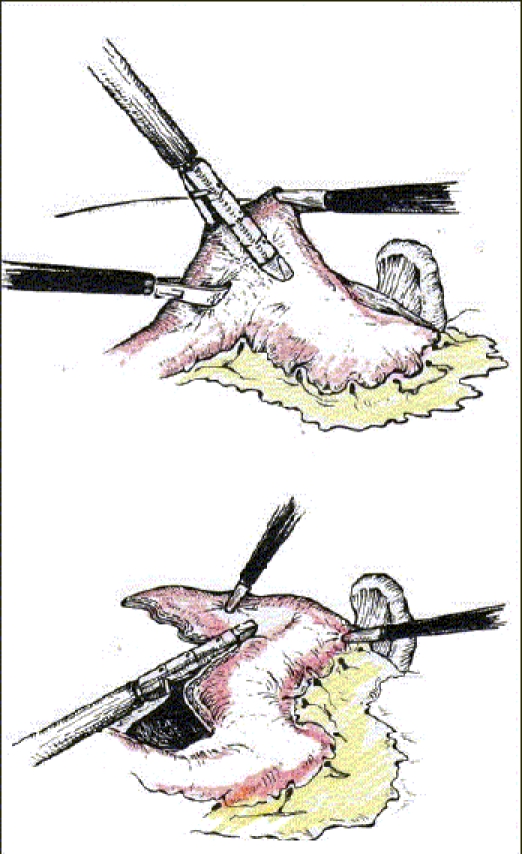
The gastric tube is created by means of the endostapler

**Figure 5B:**
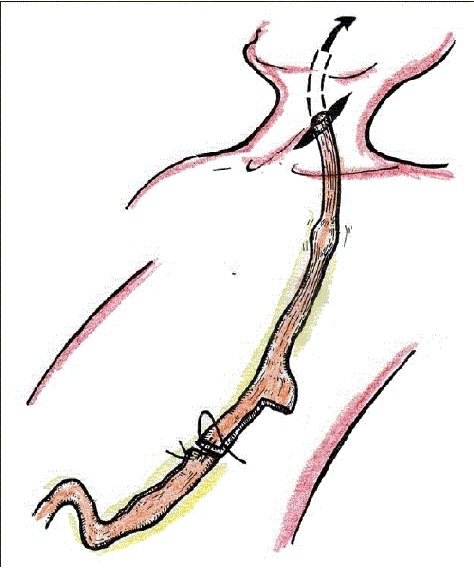
After attachment of the gastric tube to the specimen, both are retrieved through the cervical incision and the gastric tube is anastomised to the cervical esophagus

## MINIMALLY INVASIVE IVOR LEWIS ESOPHAGECTOMY

The conventional Ivor Lewis esophageal resection implies the resection of the esophagus (and lymphadenectomy) through a right thoracotomy followed by an intrathoracic anastomosis of the gastric conduit with the proximal esophagus at the level of the proximal mediastinum.[2] Potential advantages are the prevention of a cervical dissection of the esophagus and the consequent complications such as leakage, stenosis and recurrent nerve palsy. Disadvantages are the performance of an anastomosis in the thoracic cavity with all the risks of leakage with subsequent high morbidity and mortality. Initial experience with the minimally invasive Ivor Lewis is very limited. Watson *et al.*, described in 1999 a hand-assisted procedure in two patients in whom the anastomosis was performed by right thoracoscopy.[37] Nguyen *et al.*, describe a series of three patients in whom the procedure was performed completely minimally invasive. All patients had an uneventful postoperative course.[38] In 2006, the group of Pitsburgh described a series of 50 patients who underwent an Ivor Lewis procedure.[39] Indications were a short segment Barrett's esophagus with high-grade dysplasia or resectable adenocarcinoma of the gastro-esophageal junction with minimal proximal esophageal extension. Twenty-five patients received preoperatively chemotherapy or chemoradiotherapy. The planned approach included a totally laparoscopic abdominal procedure, as above described in the three stage procedure and either a right minithoracotomy or thoracoscopy. Assisted 5 cm posterolateral minithoracotomy was performed in 35 patients and in an additional group of 15 patients a near total thoracic stapled anastomosis was performed thoracoscopically. An end-to-side circular stapled anastomosis (25 mm) was performed in all patients. Operative mortality was 6%. Three patients developed an anastomotic leak; all were successfully managed conservatively. Four patients (8%) developed postoperative pneumonia. There were no recurrent laryngeal nerve injuries.

They concluded that the procedure is technically feasible. Advantages could be the minimization of the gastric mobilization, the avoidance of recurrent laryngeal injuries and that the procedure will allow a more extensive gastric resection in the case of tumor extension into the cardia in junction tumors.

## TECHNIQUE OF THE MINIMALLY INVASIVE IVOR LEWIS PROCEDURE

After double lumen intubation to collapse the right lung, the patient is placed for laparoscopic procedure. The operation starts with the laparoscopic procedure like the above mentioned technique for the three-stage procedure. Patient is then placed in a lateral decubitus position for a right thoracoscopy.

In the case of a hybrid procedure, a 5 cm posterolateral thoracotomy is performed with preservation of the serratus muscle. After mobilization of the esophagus, this is resected and an anastomosis is performed, at the level of the azygos, in a side to end fashion between the gastric conduit and the esophagus.

If ‘near total’ thoracoscopically procedure, the position of the ports are like in the above mentioned three stage procedure. Important is to enlarge the posterior inferior 8^th^ intercostal space port to 4 cm in order to introduce the circular stapler [Figure 6A]. After esophageal resection the anvil of 25 mm circular stapler is introduced into the esophagus and secured by a purse-string. The specimen is then retrieved through the well protected incision and from there the gastric conduit is exteriorized to introduce the circular stapler [Figure 6B] and complete the anastomosis [Figure 6C]. The redundant portion of the gastric conduit is then resected by endostaplers and the thoracic cavity drained.

**Figure 6A:**
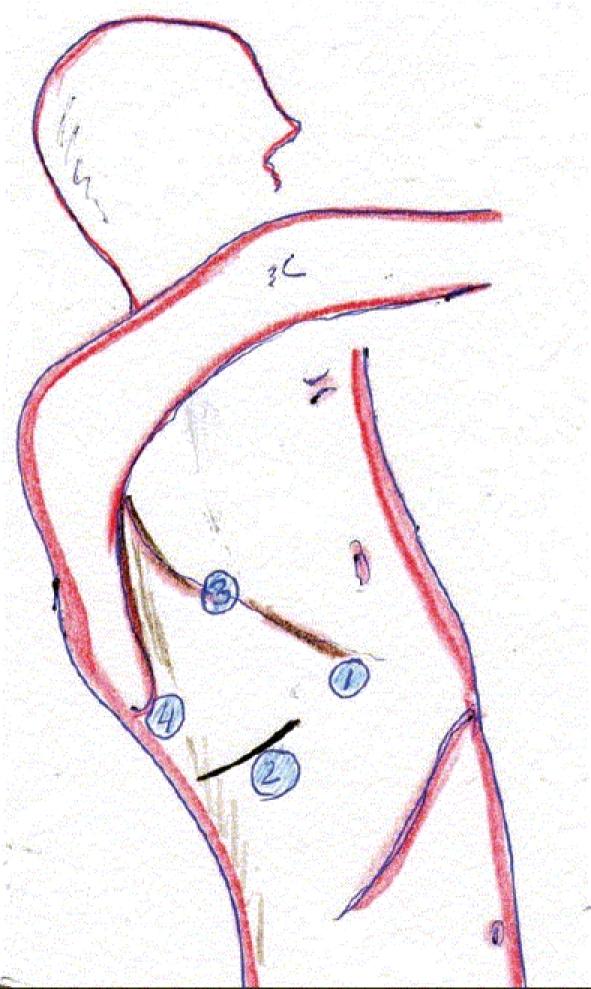
Right thoracoscopic approach for Ivor Lewis operation

**Figure 6B:**
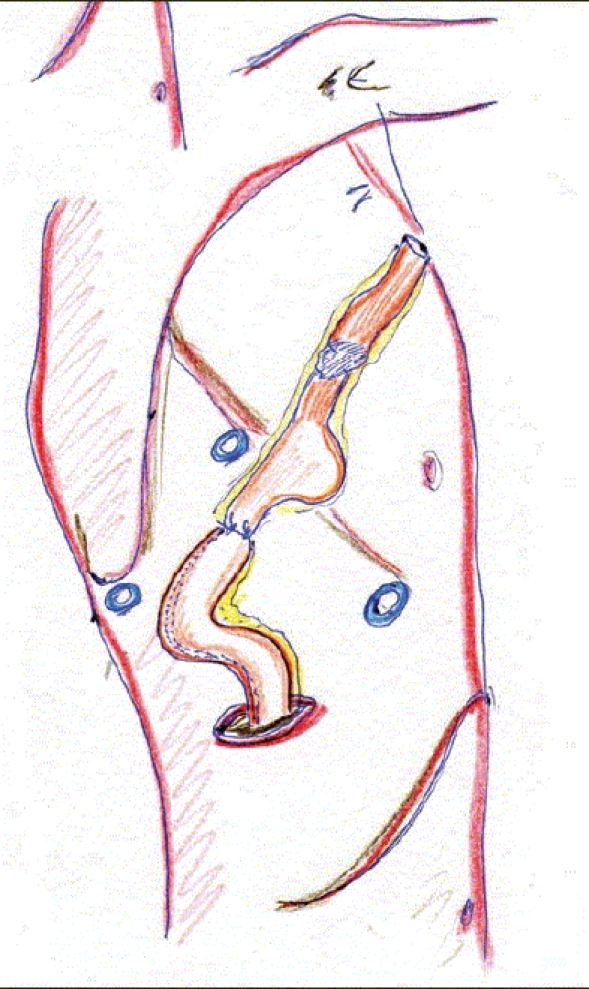
Exteriorization of the specimen through the enlarged 8th posterior trocar line (4 cm)

**Figure 6C:**
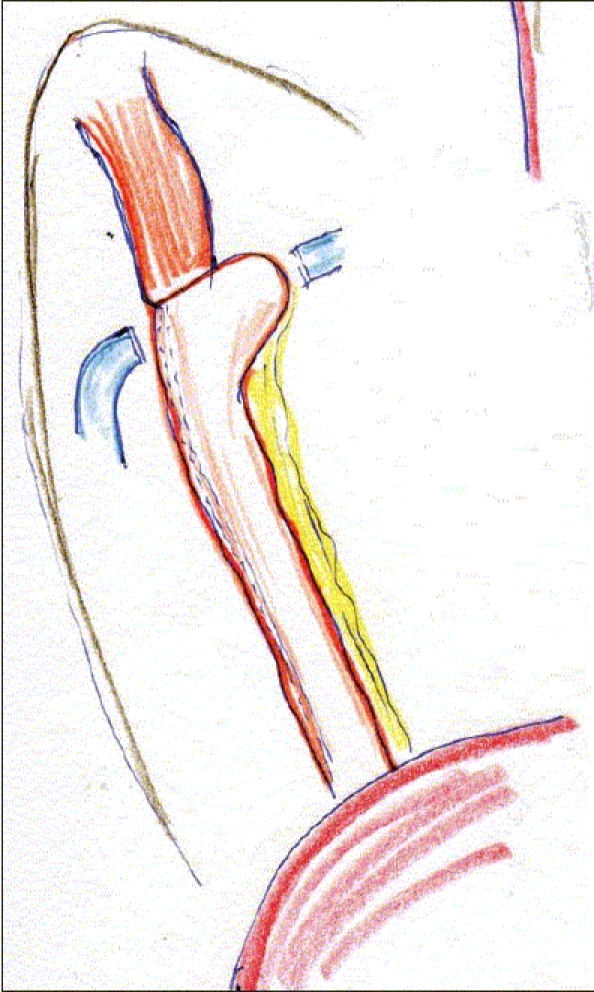
Intrathoracic anastomosis at the level of the azygos vein (end to side fashion)

## MINIMALLY INVASIVE ESOPHAGECTOMY WITH THORACOSCOPIC MOBILIZATION OF THE ESOPHAGUS IN PRONE POSITION

Cuschieri *et al.*, described in 1994 the first 6 patients operated in the prone position and compared the results with 20 patients operated in left lateral position.[17] The aim of the procedure was to avoid a total collapse of the right lung in order to avoid the frequently described postoperative pulmonary complications after total collapse of the right lung. The procedure was never again implemented until recently. Palanivelu *et al.*, from Coimbatore in India, described in 2006 a series of 130 patients, operated from 1997 to 2005, who underwent a right thoracoscopic esophagectomy in the prone position.[40] All patients had esophageal squamous cancer of the middle third of the esophagus. After dissection of the stomach and division of the esophagus at cervical level the specimen is retrieved through a minilaparotomy. Outside, the specimen is resected and the gastric conduit created and advanced into the cervical incision. Remarkable is that there were no conversions to an open procedure; mean operative time was 220 min, the median stage in the ICU was one day (range 1 to 32 days) and the median hospital stay of only eight days (4 to 68 days). Perioperative mortality was 1.5% and anastomotic leak 2.3%. Major morbidity was 12.3% (16 patients). There was no incidence of tracheal injury and a very low incidence of postoperative pneumonia.

Important was the low rate of postoperative pneumonia (1.5%), being the recurrent laryngeal palsy of 1.5%. At mean follow-up of 20 months, stage-specific survival was similar to open and other minimally invasive series. They concluded that the technique is feasible, with a low rate of pulmonary complications and less operative time.

## TECHNIQUE OF RIGHT THORACOSCOPIC ESOPHAGECTOMY IN THE PRONE POSITION

Palanivelu *et al.*, describe the operative technique in three stages.[40]

**Figure 7:**
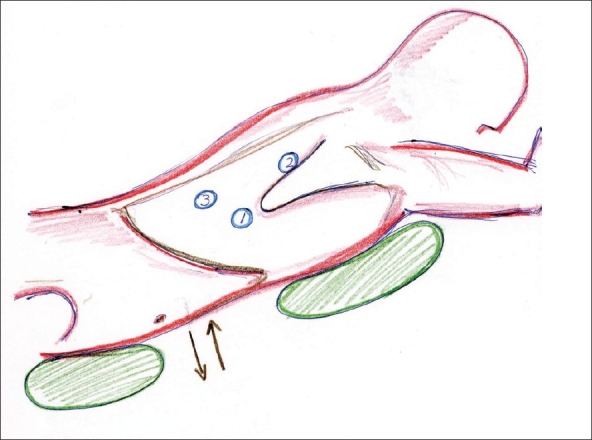
Prone position for right thoracoscopy and position of trocars according to Palanivelu *et al*.[40]

Stage 1, thoracoscopic mobilization of the esophagus and mediastinal lymphadenectomy. Patients were intubated with a single endotracheal tube (with the possibility of ventilation of both lungs by left lung ventilation with intermittent ventilation of the right lung). After intubation they were positioned in prone position leaving the abdomen free to allow respiratory movements [Figure 7].

The surgeon stands on the right side of the patient, the camera assistant to his/her left and the second assistant stands on the left side of the patient. The laparoscopic cart is positioned at the patient's left shoulder. Insufflations by Veress needle is made through puncture at the seventh intercostal space up to 6-8 mm Hg. In this manner the right lung remains collapsed through the entire operation by both, the insufflation pressure and the gravity.

A 30 degrees scope is always used. They used three ports, one of 10 and two of 5 mm, using another optional port for retraction of the lung. Port position:

7^th^ ICS below the inferior angle of the scapula (camera, 1); 5^th^ ICS 7 cm lateral from the spinous process for the right hand instruments[2] and 9^th^ ICS 7 cm lateral from the spinous process[3] for left hand instruments. A flexible upper gastrointestinal endoscope is placed in the esophagus to lift the esophagus to facilitate the dissection.

By inspection, the tumor and the surrounding anatomy are then assessed. After division of the inferior pulmonary ligament the mediastinal pleura above and below the azygos vein is opened and the vein divided. The proximal esophagus is circumferentially dissected free and encircled with a tape. By using retraction, the esophagus is dissected free from the aorta and chest wall. The thoracic duct should be preserved. The most difficult part is the dissection between the esophagus and the trachea. Mobilization of the lower third of the esophagus poses no problem. Peri-esophageal tissue and LN are included in the specimen. A complete lymphadenectomy is performed in the carinal region, from the hiatus to the carina anteriorly and of the supracarinal space. But also the space between the right bronchus and the superior vena cava and the space between left bronchus and aorta arch. In this way the entire esophagus from the thoracic outlet to the hiatus is mobilized. After leaving a drain, the lung is expanded fully and the patient's position is changed to the modified Lloyd-Davis position with anti-trendelenburg for the laparoscopic part of the operation.

Stage 2, laparoscopic mobilization of the stomach, removal of the specimen and gastric tube formation. This stage can be performed as the above mentioned three stage procedure, but Palanivelu *et al.*, exteriorized the whole specimen, after division of the cervical esophagus, through a small laparotomy, after mobilization of the stomach and lymphadenectomy of the celiac trunk. Then the specimen is resected and a gastric conduit of 5-6 cm is made along with a pyloromyotomy.

Stage 3, positioning of gastric conduit and cervical anastomosis. The gastric conduit is brought to the cervical wound and there is anastomosed to the proximal esophagus.

## COMMENT

Important advantage of this prone position approach will be the decrease of pulmonary complications. Luketich *et al.*, reports an incidence of pulmonary complications in 7.6% of the patients after right thoracoscopy with subsequent collapse of the lung. Palanivelu *et al.*, report pulmonary complications in only 1.5% of patients in their series. Possible factors to explain this are the use of a single endotracheal tube with possible two-lung ventilation. The partial ventilation of the right lung reduces the possibility of arteriovenous shunting. Moreover, in prone position the functional residual capacity is greater than in supine position. Also, ventilation-perfusion ratio is well-maintained and hypoxia and hypercarbia avoided. This reduces the extent of pulmonary dysfunction and athelectasia postoperatively. Other important advantages of the prone position are shorter anaesthesia time, decrease lung injury, excellent exposure of the operative field and a better ergonomic for the surgeon

## ROBOT ASSISTED MOBILIZATION OF THE ESOPHAGUS BY RIGHT THORACOSCOPY

**Figure 8:**
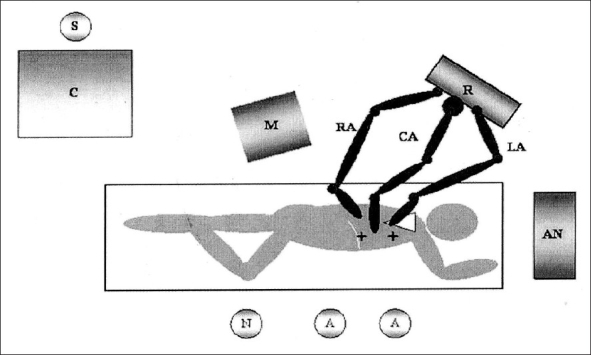
Set up and positioning of the operating team in robot-assisted thoracoscopic esophago-lymphadenectomy[13]

Horgan *et al.*, published the first report about the use of the robot to dissect the esophagus and perform a lymphadenectomy in a patient with esophageal cancer.[12] The first series of patients have been published by van Hillegersberg *et al.* in 2006 [Figure 8].[13] They report a series of 21 patients who underwent a RTE (robot-assisted thoracoscopic esophagectomy) using the Da Vinci robotic system. A total of 18 (86%) procedures were completed thoracoscopically. Afterwards through a median laparotomy the gastric stage of the operation was performed. The operating time for the thoracoscopic phase was 180 min (120-240 min) being the median blood loss 400 ml range 150-700 ml. Median ICU was four days (91-129 days) and hospital stay was 18 days (11-182 days). Pulmonary complications occurred in 10 patients (48%) and one patient (5%) died of a tracheoesophageal fistula. A median of 20 (9-30) lymph nodes were retrieved.

The authors conclude that RTE was feasible, providing an effective lymphadenctomy with low blood loss. Standardization of the technique should reduce the complication rate, which is in the range of the rate for open resection.

## DISCUSSION

Surgical treatment of esophageal cancer, with important dissections at abdominal, thoracic and cervical spaces means a tremendous operative trauma for the patient with high postoperative discomfort and a high morbidity and mortality. The approach of the esophagus through a right thoracotomy in combination with a laparotomy and cervical incision has an important rate of complications, especially the pulmonary, that will account for the long intensive care stay. Transhiatal approach, according to Orringer will reduce this complication rate by avoiding opening of thoracic cavity and therefore reducing the number of pulmonary complications. Drawback of this approach is the blind character of the mediastinal dissection that can only be performed with some displacement of the heart with consequent hemodynamic repercussions. A randomized study from Hulscher *et al.*, comparing the standard transhiatal (and lymphadenectomy of the celiac trunk) approach with the transthoracic approach (and lymphadenectomy of the mediastinum and celiac trunk) has not demonstrated a significant difference between the groups concerning survival at five years. An important trend exists in favour of the transthoracic group, but not for the tumors located in the distal esophagus and cardia. In order to increase visualization during the mediastinal dissection of the esophagus decreasing the operative trauma and postoperative complications, different minimal invasive approaches have been developed first as imitation of the conventional procedures: the three stage procedure (right thoracoscopy, laparoscopy and cervical anastomosis), the Ivor-Lewis procedure (laparoscopy, right thoracoscopy and thoracic anastomosis) and the transhiatal approach. Later on, but also initiated early in the nineties by Cuschieri, the right thoracoscopic approach in prone position is becoming increasingly used because the reduction of postoperative pulmonary complications. In the beginning, all procedures had long operative times and the postoperative complications rates were similar to the conventional approach. More experience of surgeons with advanced minimally invasive procedures, the introduction of more sophisticated devices for dissection and sealing of tissues, such as Ligasure, Ultracision and endostaplers and the general acceptance that oncological procedures can be performed by minimally invasive procedures without oncological disadvantage for the patient, has stimulated importantly further developments. The enthusiasm of different Japanese groups and the systematically standardization of the Pittsburgh's group of Luketich have demonstrated that the three stage procedure can be performed not only safely, but also in an comparable operating time with important advantages in the postoperative recovery of the patients and a good oncological outcome, at least as good as after conventional surgery. One of the problems of this approach will be the difficulty of the double endotracheal tube and the collapse of the right lung to visualize adequately the mediastinum. With short operative time, the produced shunt by the collapse is to overcome; nevertheless in elderly people and longer thoracoscopic time, pulmonary complications may rise. Luketich *et al.*, described an incidence of 7.7% of pneumonia and 1,8% of acute respiratory disease as major complications along with 4.5% atelectasis with mucus plug requiring bronchoscopy as minor complications. Moreover these problems do not affect the hospital stay of seven days and the very low mortality rate of 1.4%. To overcome these complications, the right thoracoscopic esophageal dissection performed in prone position will permit a reasonable partial ventilation of the right lung with as consequence less postoperative pulmonary complications. Palanivelu *et al.*, have an incidence of 1.54% of pneumonia and 0.77% of acute respiratory disease. Moreover their operative time is comparable with the standard conventional and right thoracoscopic procedures. Concerning oncological parameters, R0 -R1 resections, number of lymph nodes resected and finally the survival according to stage, the two most important series of Luketich *et al.* and Palanivelu *et al.*, are similar to the published with conventional procedures. The role of the promising robotic assisted esophageal resection has to be defined in a near future.

Smithers *et al.*, compares recently the outcomes between open and minimally invasive esophagectomy and concluded that minimally invasive techniques to resect the esophagus in patients with cancer were confirmed to be safe and comparable to an open approach with respect to postoperative recovery and cancer survival.[41]

Another important point is to define the role of the laparoscopic transhiatal approach. Important advantage will be the avoidance of the thoracoscopic stage with theoretically less respiratory complications. As for the conventional transhiatal, the approach is ideal for the very distal esophageal and gastro-esophageal junction tumors. These tumors are the most frequent in the western world. Important advantage for this approach will be the initial assessment of the abdomen for metastases, the dissection of the tumor between distal esophagus and stomach, with a good view of both sides of the tumor, the hiatus and both pleuras. The dissection can be accomplished very adequately in the anatomical planes up to the carina. The dissection and construction of the gastric conduit will be performed at the same stage without changing the positioning of the patient. Drawback of this procedure is the lack of carinal lymphadenectomy. The results obtained by our group with this approach in an unselected group of 50 patients are very promising[42] with a similar survival as in the conventional approach.

Concerning the survival of the patients, new protocols will give more insight in the role of chemoradiation as neoadjuvant treatment for these tumors. Probably the combination of a preoperative precise stagimg of the tumor, the use of efficacious neoadjuvant therapy and the minimally invasive surgical resection will be the adequate approach for these patients, producing less postoperative complications and a better survival.
